# Biogeography and Population Divergence of Microeukaryotes Associated with Fluids and Chimneys in the Hydrothermal Vents of the Southwest Indian Ocean

**DOI:** 10.1128/spectrum.02632-21

**Published:** 2022-09-19

**Authors:** Yue Zhang, Ning Huang, Hongmei Jing

**Affiliations:** a CAS Key Laboratory for Experimental Study under Deep-Sea Extreme Conditions, Institute of Deep-Sea Science and Engineering, Chinese Academy of Sciences, Sanya, China; b HKUST-CAS Sanya Joint Laboratory of Marine Science Research, Chinese Academy of Sciences, Sanya, China; c Southern Marine Science and Engineering Guangdong Laboratory (Zhuhai), Zhuhai, China; Nanyang Technological University

**Keywords:** microeukaryotes, hydrothermal vents, high-throughput sequencing, parasitic, distribution

## Abstract

Deep-sea hydrothermal vents have been proposed as oases for microbes, but microeukaryotes as key components of the microbial loop have not been well studied. Based on high-throughput sequencing and network analysis of the 18S rRNA gene, distinct biogeographical distribution patterns and impacting factors were revealed from samples in the three hydrothermal fields of the southwest Indian Ocean, where higher gene abundance of microeukaryotes appeared in chimneys. The microeukaryotes in the fluids might be explained by hydrogeochemical heterogeneity, especially that of the nitrate and silicate concentrations, while the microeukaryotes in the chimneys coated with either Fe oxides or Fe-Si oxyhydroxides might be explained by potentially different associated prokaryotic groups. Population divergence of microeukaryotes, especially clades of parasitic Syndiniales, was observed among different hydrothermal fluids and chimneys and deserves further exploration to gain a deeper understanding of the trophic relationships and potential ecological function of microeukaryotes in the deep-sea extreme ecosystems, especially in the complex deep-sea chemoautotrophic habitats.

**IMPORTANCE** Deep-sea hydrothermal vents have been proposed as oases for microbes, but microeukaryotes as key components of the microbial loop have not been well studied. Based on high-throughput sequencing and network analysis of the 18S rRNA gene, population divergence of microeukaryotes, especially clades of parasitic Syndiniales, was observed among different hydrothermal fields. This might be attributed to the hydrogeochemical heterogeneity of fluids and to the potentially different associated prokaryotic groups in chimneys.

## INTRODUCTION

Microeukaryotes play key ecological roles in both benthic and pelagic marine ecosystems by mediating the transfer of organic matter and energy between different trophic levels (1, 2). Depending on the presence of organic debris and prokaryotic cells, heterotrophic eukaryotes dominate the deep-sea ecosystems and tend to spread in the direction of nutrient sources (3) and integrate the microbial loop into classical marine food webs by transferring dissolved organic matter utilized by heterotrophic bacteria to higher trophic levels (4). They are also grazers that control the populations of prokaryotes in the hydrothermal vents and cold seeps. For instance, ciliates (Alveolata) actively grazed on microbial mats adjacent to cold seeps (5). Many microeukaryotes developed parasitic and symbiotic relationships with animals or prokaryotes to adapt to anoxic environments (6, 7). Gregarine parasites (Excavata) have been reported to inhabit anoxic biospheres (6). Ciliates have developed symbiotic relationships with archaea and bacteria to adapt to anoxic environments (7, 8). Variation in metabolic status together with high morphological and genetic diversity enables microeukaryotes to fulfill diverse roles in different marine microbial ecosystems (1).

Hydrothermal vents occur along divergent plate boundaries in every ocean and are commonly located at midocean ridges worldwide (9). Heat and chemical species are transferred from the lithosphere to the ocean and have an influence on ocean chemistry and microbiology (10, 11). Since the first discovery of hydrothermal vents in the late 1970s, vent-associated microorganisms have attracted the attention of scientists. Among these microorganisms, chemosynthetic bacteria and archaea form the foundation of vent ecosystems and have been extensively described (12, 13). Diverse and abundant macro-organisms such as shrimps, mussels, crabs, and gastropods around hydrothermal structures have also been commonly observed (14, 15). However, very few studies have been devoted to the microeukaryotes associated with deep-sea vents (16–20).

By far, studies on hydrothermal microeukaryotes have been mainly limited to the microscopy observation of ciliates at East Pacific Rise vents (18), the isolation and culture of thermophilic ciliates from the hydrothermally heated sea floor at Vulcano Island in Italy (19), and the microeukaryotic taxonomy composition and ecological roles in hydrothermal vents of Guaymas Basin (20) and Lost City (16) based on next-generation sequencing. The above-described studies focused on one specific hydrothermal vent, and a comparison of fluids and chimneys was absent; therefore, a comprehensive analysis of microeukaryotes based on fluids, chimneys, and seafloor sulfide deposits from different hydrothermal vents will help to gain a full picture of those microbial groups and might provide insights into their adaptation in the extreme deep-sea ecosystems.

The Southwest Indian Ridge (SWIR) is a highly oblique ultraslow-spreading ridge, extending over 7,700 km between the Bouvet and Rodriguez triple junctions (21). The first active hydrothermal field was discovered at 49°39′E in 2007, and subsequently, several active hydrothermal fields and hydrothermal anomalies were found between 49°E and 63°E along the SWIR (22, 23). Prokaryote (24) and macro-organism (14) inhabitants have been widely reported. However, little is known about the diversity and distribution of microeukaryotes at those hydrothermal vents. Aided by the development of submersibles, we conducted a molecular assay to characterize the biodiversity and potential trophic relationships of the microeukaryotes by analysis of fluids and chimneys from three hydrothermal fields (i.e., Longqi, Kairei, and Edmond) to lay a foundation for future investigations regarding the biogeography and possibly environmental adaptation of microeukaryotes in deep-sea ecosystems.

## RESULTS

### Hydrographic conditions.

Hydrothermal venting fluid and chimney samples were collected from the Longqi, Kairei, and Edmond hydrothermal fields in the Indian Ocean (see Fig. S1 in the supplemental material). Longqi is located on a high mound on the southeast wall of the ridge valley, at a water depth of ~2,760 m in the southwest Indian Ocean (22). In Longqi, samples were collected from Vent S, which is spread over a 120- by 100-m^2^ area with dense hydrothermal faunas, such as mussels, scaly-foot gastropods, stalked barnacles, and sea anemones (25). Kairei and Edmond, located along the Central Indian Ridge, show an intermediate spreading rate (~48 mm year^−1^). These two hydrothermal fields show geochemically distinct signatures in their vent fluids, despite being only 160 km apart (26). Kairei is located at a 20-km-long ridge segment immediately north of the Rodrigues Triple Junction and characterized by an H_2_-rich-fluid-bearing vent at a depth of ~2,420 to 2,460 m (27). The active vent site extends about 80 m along the rift wall, which is about 30 m wide (26, 28). Edmond is located at a depth of ~3,270 to 3,320 m on a small protrusion from the eastern rift valley wall and has higher concentrations of CH_4_ and CO_2_ than those in Kairei (29). The main area of the active vent field is about 40 m^2^ (29).

The NO_2_^−^ concentrations in the fluids of the three hydrothermal fields were generally similar, and the lowest concentration of NO_3_^−^ was found in fluid sample SY110_F2 (Table 1). The concentrations of PO_4_^3−^ varied greatly at different stations and in different hydrothermal vents, with the maximum occurring in SY091-F1 in Longqi. The SiO_3_^2−^ concentration was significantly higher in the Edmond vent than in the other two vents.

**TABLE 1:** Environmental parameters of the fluids and characteristics of the chimney samples collected from the hydrothermal vents in the southwest Indian Ocean

Location	Depth (m)	Fluid sample^*a*^	Fluid sample characteristics				Chimney sample^*b*^	Chimney description	Associated organism(s)
			NO_3_^−^ (μg/L)	PO_4_^3−^ (μg/L)	SiO_3_^2−^ (μg/L)	NO_2_^−^ (μg/L)			
Longqi	2,775	SY096_F1	12.03	13.75	561.99	2.98	SY093_G3	Active chimney coated with Fe oxides	None
		SY096_F2	12.17	5.34	593.74	3.07			
		SY096_F3	12.08	6.86	605.79	3.09	SY094_G1	Inactive chimney coated with Fe oxides	Mussel
		SY096_F4	11.99	6.18	622.35	3.12			
		SY110_F1	9.89	10.12	638.19	3.00	SY099_G1	Active chimney coated with Fe oxides	Snail, mussel, shrimp
		SY110_F2	8.47	7.65	463.84	3.12			
		SY110_F3	12.20	7.97	660.82	3.53	SY105_G6	Active chimney coated with Fe oxides	Mussel, Nameplates
		SY110_F4	12.05	11.22	1,005.06	3.18			
Kairei	2,450	SY136_F1	11.98	6.72	1,063.62	3.18	SY136_G6	Inactive chimney coated with Fe-Si oxyhydroxides	Actiniaria
		SY136_F2	11.97	6.17	1,094.93	3.16	SY136_G7	Inactive chimney	None
		SY136_F3	11.95	6.59	1,092.17	3.28	SY136_G8	Seafloor sulfide deposits	None
		SY136_F4	12.24	9.35	1,331.62	3.59	SY143_G4	Inactive chimney coated with Fe oxides	None
Edmond	3,284	SY148_F1	11.69	10.06	1,227.72	2.97	SY139_G3U/B	Inactive chimney coated with Fe-Si oxyhydroxides	None
							SY139_G8	Seafloor sulfide deposits (Fe-Si oxyhydroxides)	Actiniaria
		SY148_F2	11.52	10.83	1,218.75	3.21	SY139_G11	Seafloor sulfide deposits	Actiniaria
							SY148_G3	Active chimney coated with Fe-Si oxyhydroxides	Shrimp
		SY148_F3	11.63	8.68	1,372.84	3.29	SY148_G5	Seafloor sulfide deposits (Fe-Si oxyhydroxides)	None
							SY150_G6	Seafloor sulfide deposits	None

a F represents fluid.

b G represents chimney.

In addition, 14 chimney samples (including five seafloor sulfide deposits) were collected from three hydrothermal fields. Chimney samples SY136_G8, SY139_G8, SY139_G11, SY148_G5, and SY150_G6 were dark overall but coated with yellow and brown patches on their top portions (Table 1). Subsequently, chimney sample SY139_G8 was divided into two sections for a comparison between the upper and lower parts of the chimney. Numerous mussels were attached to the exterior of chimney samples SY094_G1, SY099_G1, and SY105_G6; Actiniaria and shrimp were also common attachments to chimney samples SY136_G6, SY139_G8, SY139_G11, and SY148_G3. The detailed features of chimneys can be found in Table 1.

### Community diversity and gene abundance.

**FIG 1:**
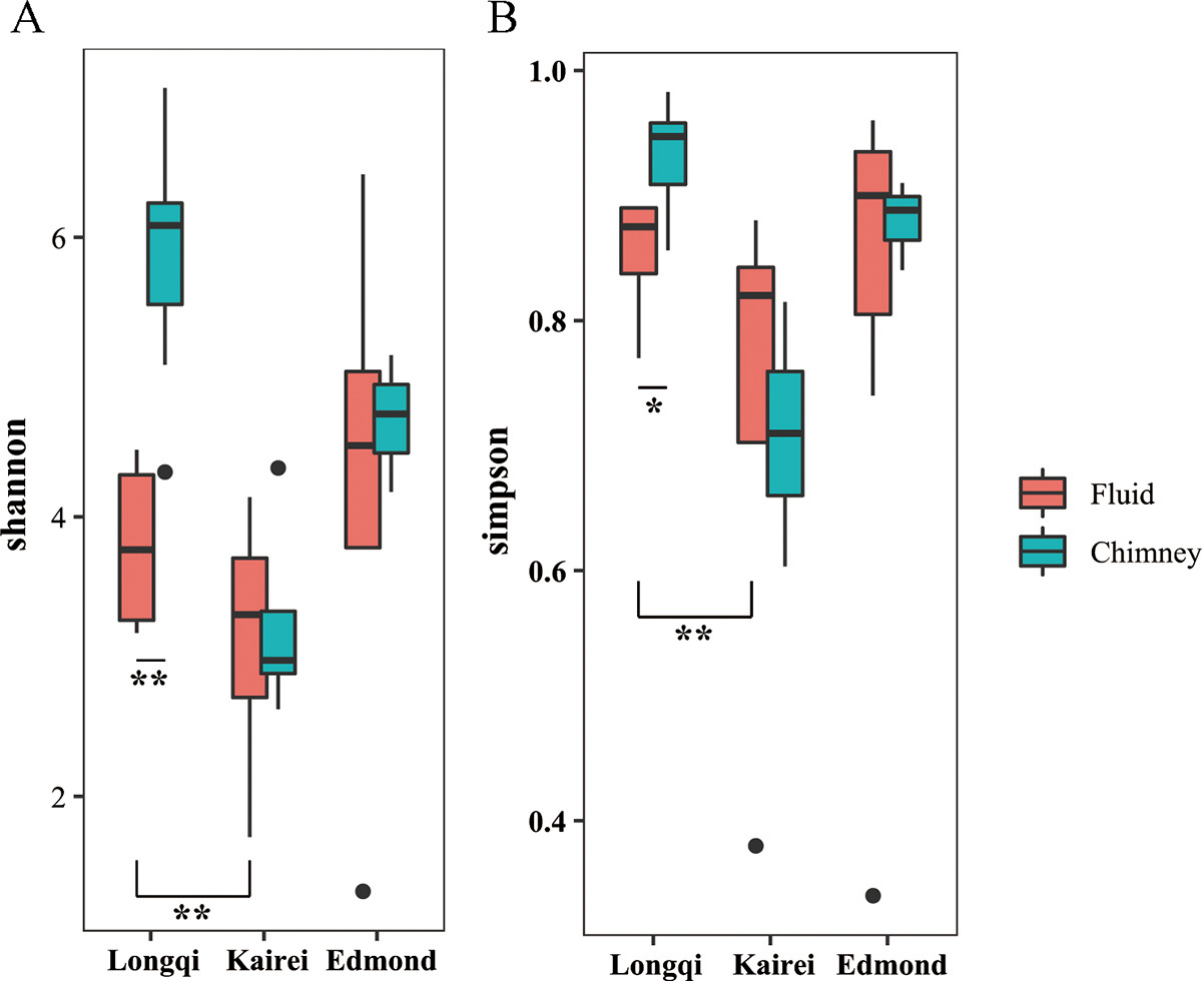
Shannon (A) and Simpson (B) indices of microeukaryotes among the fluids and chimneys in different hydrothermal vents in the southwest Indian Ocean. **, *P < *0.01; *, *P < *0.05.

In total, 824,901 and 580,967 high-quality sequences were generated from the fluids and chimneys, respectively (Table S1). Amplicon sequence variants (ASVs) totaling 6,034 and 1,189 were generated from quality reads. The maximum and minimum numbers of ASVs were found in fluid sample SY096_F1 and chimney sample SY099_G1, respectively. As for community diversity, a significant difference between Longqi and Kairei was observed in the fluid and chimney samples for Shannon (Fig. 1A) and Simpson (Fig. 1B) indexes. The highest diversity of the Shannon and Simpson indices among all samples occurred in fluid sample SY096.

**FIG 2:**
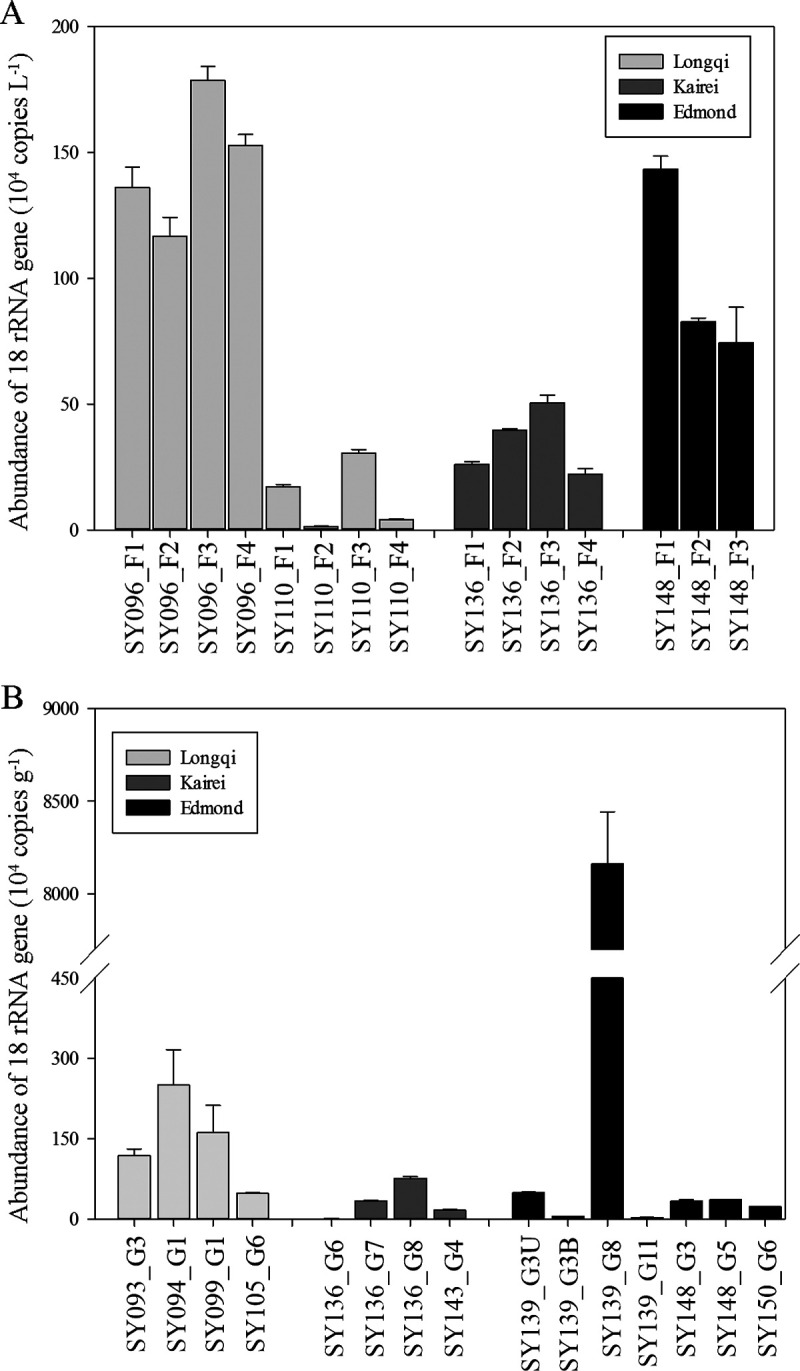
18S rRNA gene abundances of microeukaryotes in the fluids (A) and chimneys (B) in different hydrothermal vents in the southwest Indian Ocean. The error bars represent standard deviations of triplicate reactions for each sample.

In addition, the gene abundance of microeukaryotes determined by quantitative PCR (qPCR) was slightly higher in the chimneys (~10^4^ to 10^7^ copies/g) than in the fluids (~10^4^ to 10^6^ copies/L) (Fig. 2). Considering the samples in the same hydrothermal vent as a whole, the gene abundance of microeukaryotes in the Longqi vent was the highest for both fluid and chimney samples. For chimney samples, the gene abundance in chimney sample SY139_G8 was the highest.

### Community composition of microeukaryotes.

**FIG 3:**
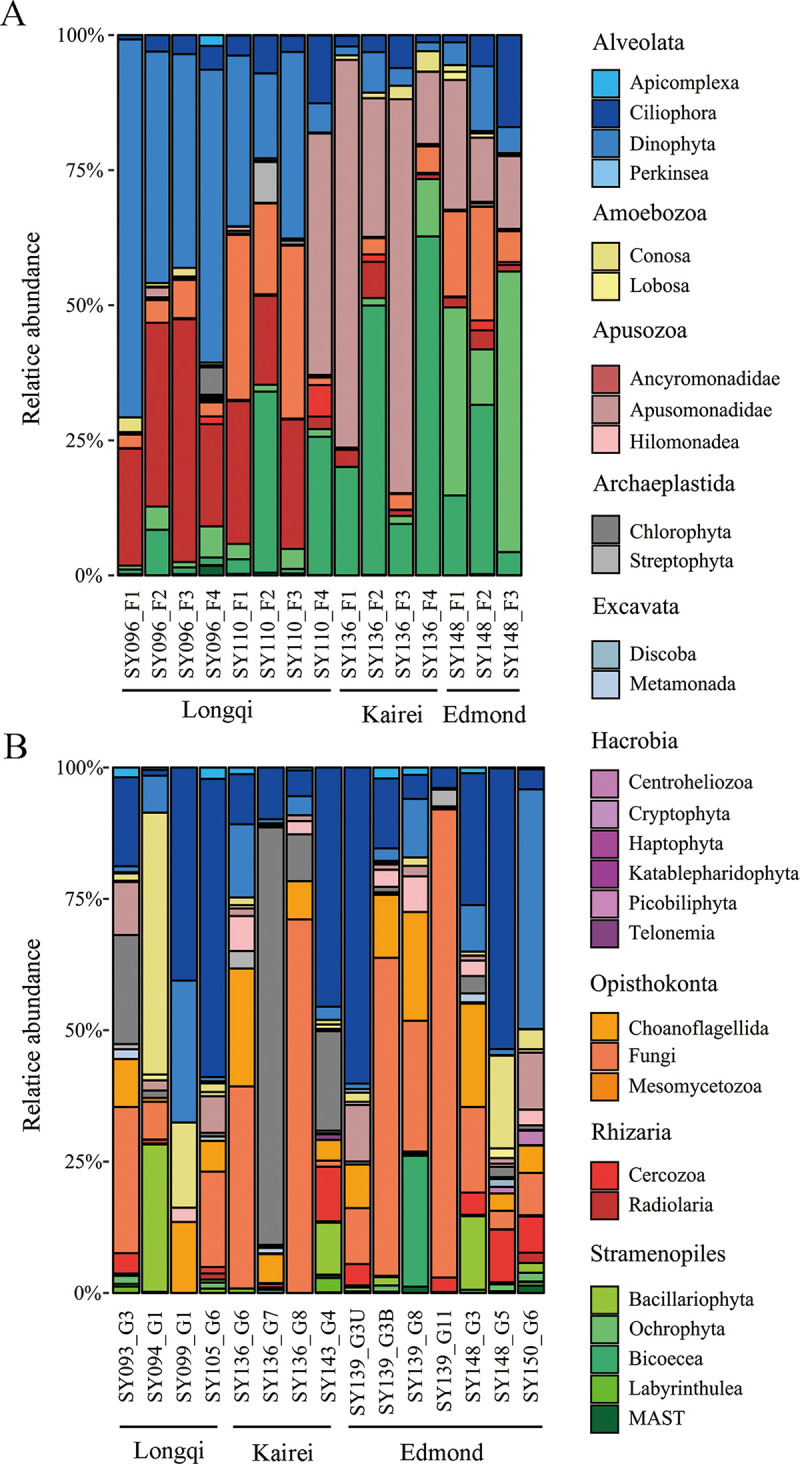
Community structure of microeukaryotes of the fluids (A) and chimneys (B) in different hydrothermal vents in the southwest Indian Ocean at the level of supergroups.

For the fluids, Alveolata and Rhizaria dominated in Longqi, and their relative abundance was higher in SY096 samples than in SY110 samples, with a higher proportion of Fungi found in the latter (Fig. 3A). Stramenopiles and Apusozoa (mainly Apusomonadidae) were predominant in Edmond and Kairei, and higher proportions of stramenopiles (mainly Ochrophyta) and Fungi occurred in the former. As for the chimney samples, most of them were dominated by Alveolata and Opisthokonta (Fig. 3B). The relative abundance of Archaeplastida was higher in Kairei than in the other vents. Non-oxide-attached chimneys were generally composed of microbial communities with low diversity, while chimneys coated with Fe oxides tended to have higher proportions of Alveolata; chimneys with Fe-Si oxyhydroxides tended to have higher proportions of Opisthokonta (mainly Fungi) and Apusozoa (mainly Hilomonadea). In addition, photoautotrophic groups, such as Chlorophyta, Streptophyta, and Bacillariophyta, were detected in deep-sea hydrothermal vents as well.

**FIG 4:**
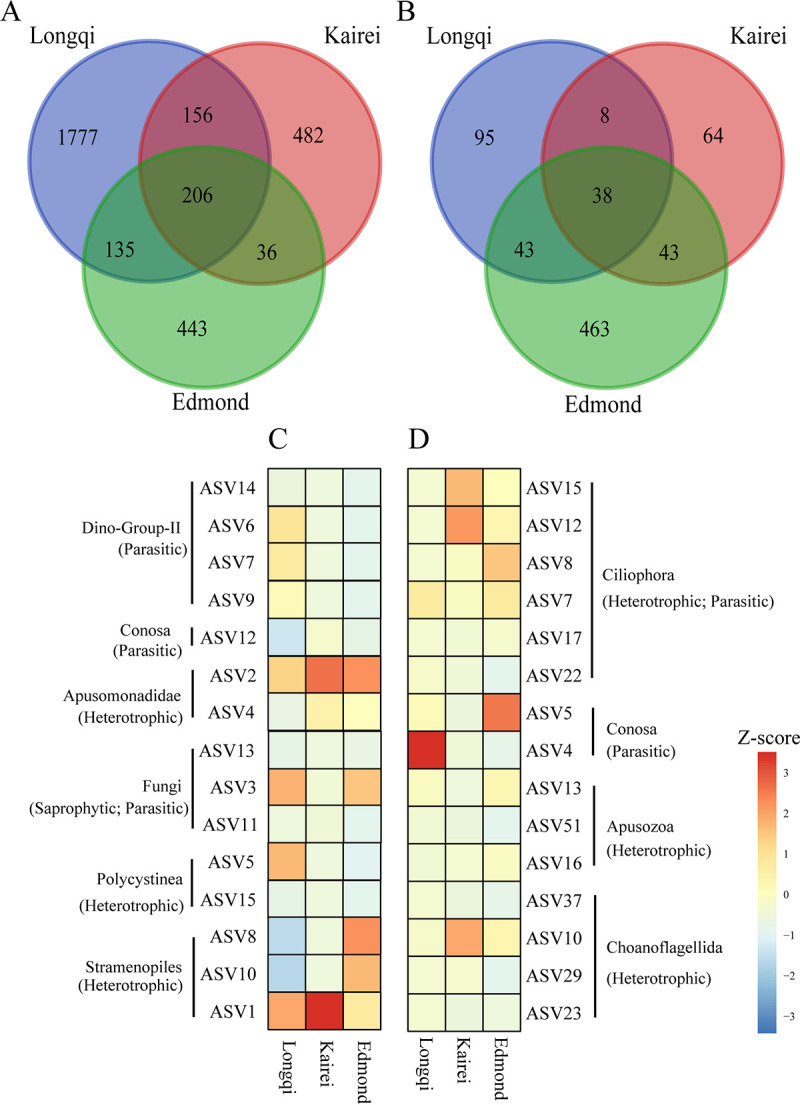
Venn diagrams representing the overlap of total ASVs in the fluids (A) and chimneys (B) and Z-score heat map boxes demonstrating the community compositions based on shared ASVs with a minimum of 1% of total sequences for fluids (C) and chimneys (D) in different hydrothermal vents of the southwest Indian Ocean.

Venn diagrams illustrating the overlap of ASVs were plotted to show the similarities among different sampling sites. A higher percentage of shared ASVs among the three hydrothermal fields occurred in the fluids (6.37%) than in the chimneys (5.04%) (Fig. 4A). In terms of specific ASVs, the highest numbers were found in Longqi for fluids (i.e., 1,777 ASVs) (Fig. 4A) and in Edmond for chimneys (i.e., 463 ASVs) (Fig. 4B). A heat map plot based on the top 15 most common ASVs demonstrated that more Dino-Group I, Dino-Group II, Polycystinea, Fungi, and stramenopiles were found in the fluids (Fig. 4C), while Ciliophora, Apusozoa, and Choanoflagellida were major groups in the chimneys (Fig. 4D).

### Environmental effects.

**FIG 5:**
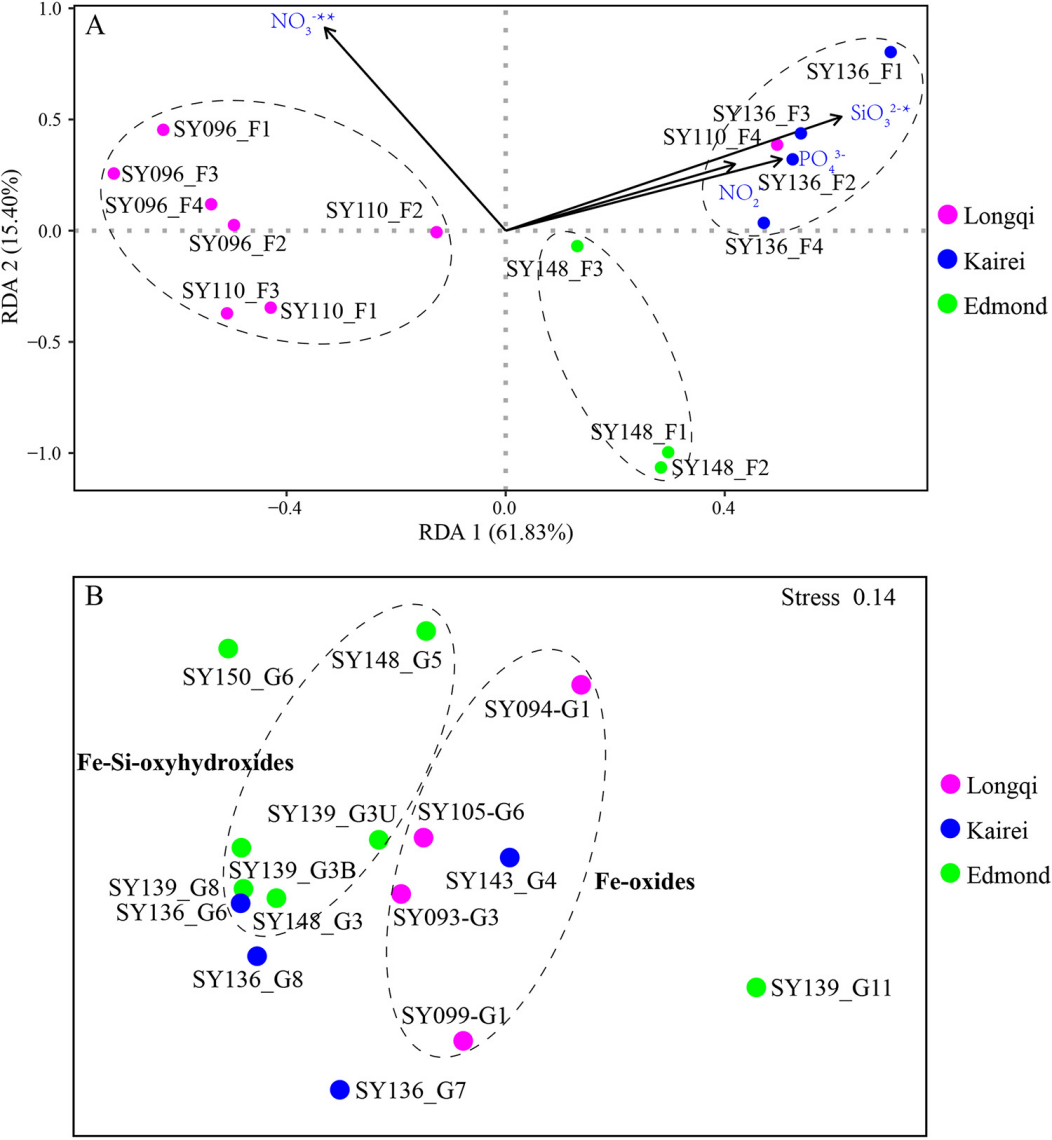
RDA of microeukaryotic communities in fluids (A) and nMDS plot based on the total ASVs in chimneys (B) in different hydrothermal vents in the southwest Indian Ocean.

Redundancy analysis (RDA) based on the microeukaryotic communities in fluids with associated environmental parameters demonstrated that NO_3_^−^ and SiO_3_^2−^ were the key environmental parameters that significantly influence the fluid eukaryotic community structure (*ρ* < 0.01 and *ρ* < 0.05, respectively) (Fig. 5A). The first two ordination axes of RDA explained 77.23% of the total variance. In addition, fluids from each vent formed a distinct cluster, except for fluid sample SY110_F4. As for the chimney samples, nonlinear multidimensional scaling (nMDS) analysis showed that chimneys coated with Fe-Si oxyhydroxides and those coated with Fe oxides formed two distinct clusters, while chimney samples (SY136_G7, SY136_G8, SY139_G11, and SY150_G6) without obvious organisms attached were scattered (Fig. 5B).

### Phylogenetic trees.

Maximum-likelihood (ML) phylogenetic trees were constructed based on the top 50 ASVs in fluids (see Fig. S2 in the supplemental material) and chimneys (Fig. S3), respectively. In the ML tree for fluids, most ASVs had a higher abundance in Longqi. ASVs were closely linked to phylotypes from the bathypelagic waters or surface sediments of the deep sea and were associated with parasitic (mainly Syndiniales), saprophytic (mainly Fungi), and heterotrophic (mainly stramenopiles and Rhizaria) assemblages. ASV20, with a higher abundance in Longqi, was closely linked to phylotypes from hydrothermal vents; ASV48, with a higher abundance in Edmond, was closely linked to phylotypes from a deep hydrothermal habitat. ASV28, with a higher abundance in Longqi, was closely linked to phylotypes from anoxic marine environments.

As for the ML tree for chimneys, most ASVs had a higher abundance in Edmond. ASVs were generally affiliated with heterotrophic clusters (mainly Choanoflagellatea, Apusomonadidae, and Cercozoa), containing phylotypes from extreme environments, such as anoxic basins and hydrothermal vents. ASV15, with a higher abundance in Edmond, was closely linked to phylotypes from the deep-sea cold seeps; ASV20, with a higher abundance in Longqi, was closely linked to phylotypes from a shallow-water hydrothermal system; and ASV47, with a higher abundance in Kairei, was closely linked to phylotypes from deep-sea hydrothermal vents.

### Cooccurrence network.

**FIG 6:**
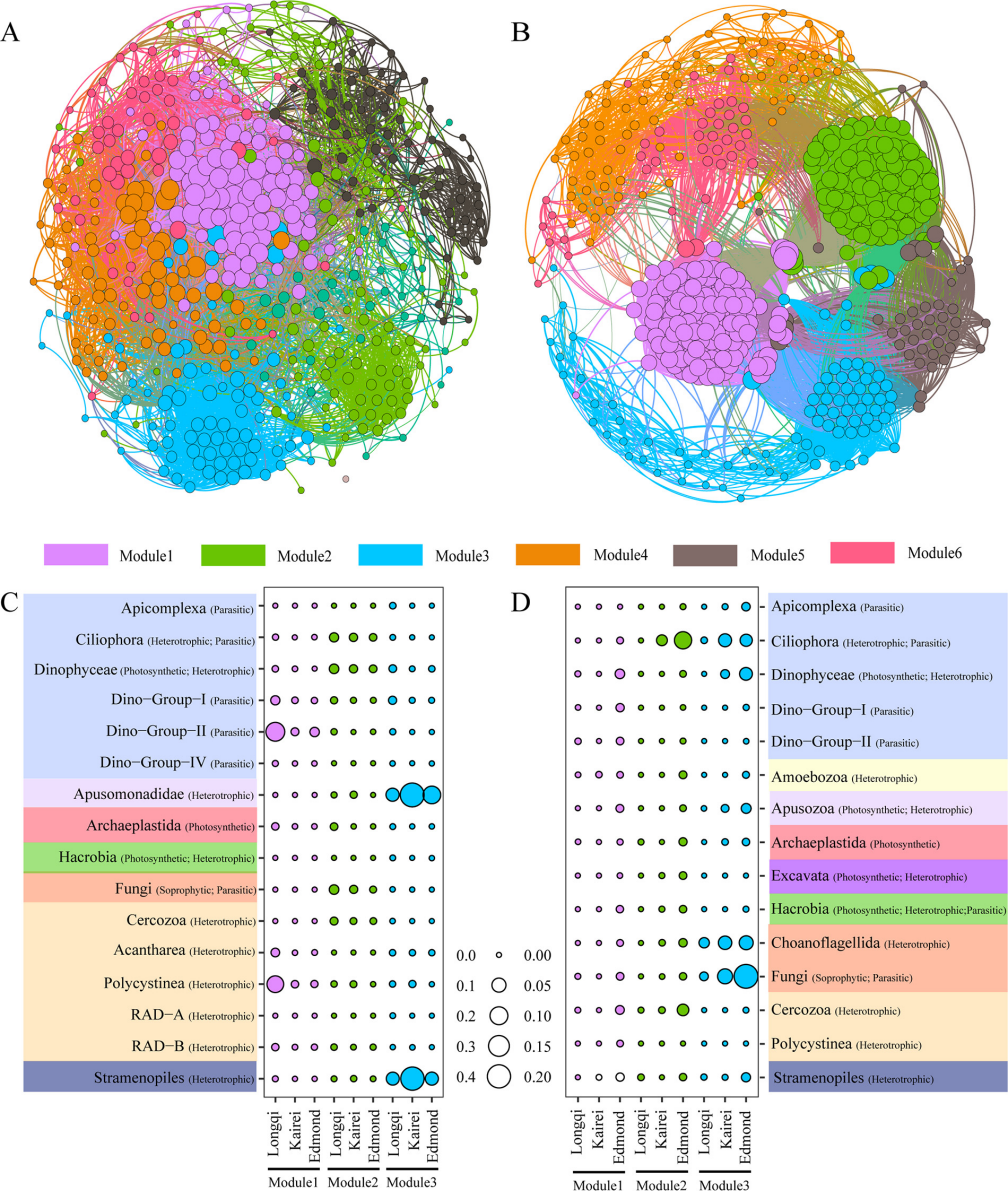
Networks of cooccurring microeukaryotic taxa (ASV > 0.001) based on a correlation analysis in fluids (A) and chimneys (B). The proportion of each bubble was based on the relative abundance of the ASV categories in fluids (C) and chimneys (D) in different hydrothermal vents in the southwest Indian Ocean.

To elucidate the interactions between the microeukaryotic groups in different vents, network analyses were conducted based on ASVs with sequence abundances greater than 0.001 for fluids and chimneys. For the fluids, the resulting network consisted of 498 nodes and 22,017 edges, and the modularity index was 0.585 (Fig. 6A). As the modularity index was >0.4, this suggests that the network had a modular structure (30). For the chimneys, the resulting network consisted of 478 nodes and 8,904 edges, and the modularity index was 0.601 (Fig. 6B). In total, six major modules with a correlation of 0.60 were defined in fluids and chimneys.

A bubble plot was further made for the three major modules in, respectively, fluids and chimneys. For the fluids, module 1 was mainly composed of parasitic types (e.g., Dino-Group II), and module 3 was mainly composed of heterotrophic types (e.g., Apusomonadidae and stramenopiles) (Fig. 6C). For chimneys, module 2 was mainly composed of heterotrophic types (e.g., Ciliophora and Cercozoa), while module 3 was mainly composed of heterotrophic and saprophytic types (e.g., Fungi and Choanoflagellida) (Fig. 6D).

### Clades of parasitic Syndiniales.

**FIG 7:**
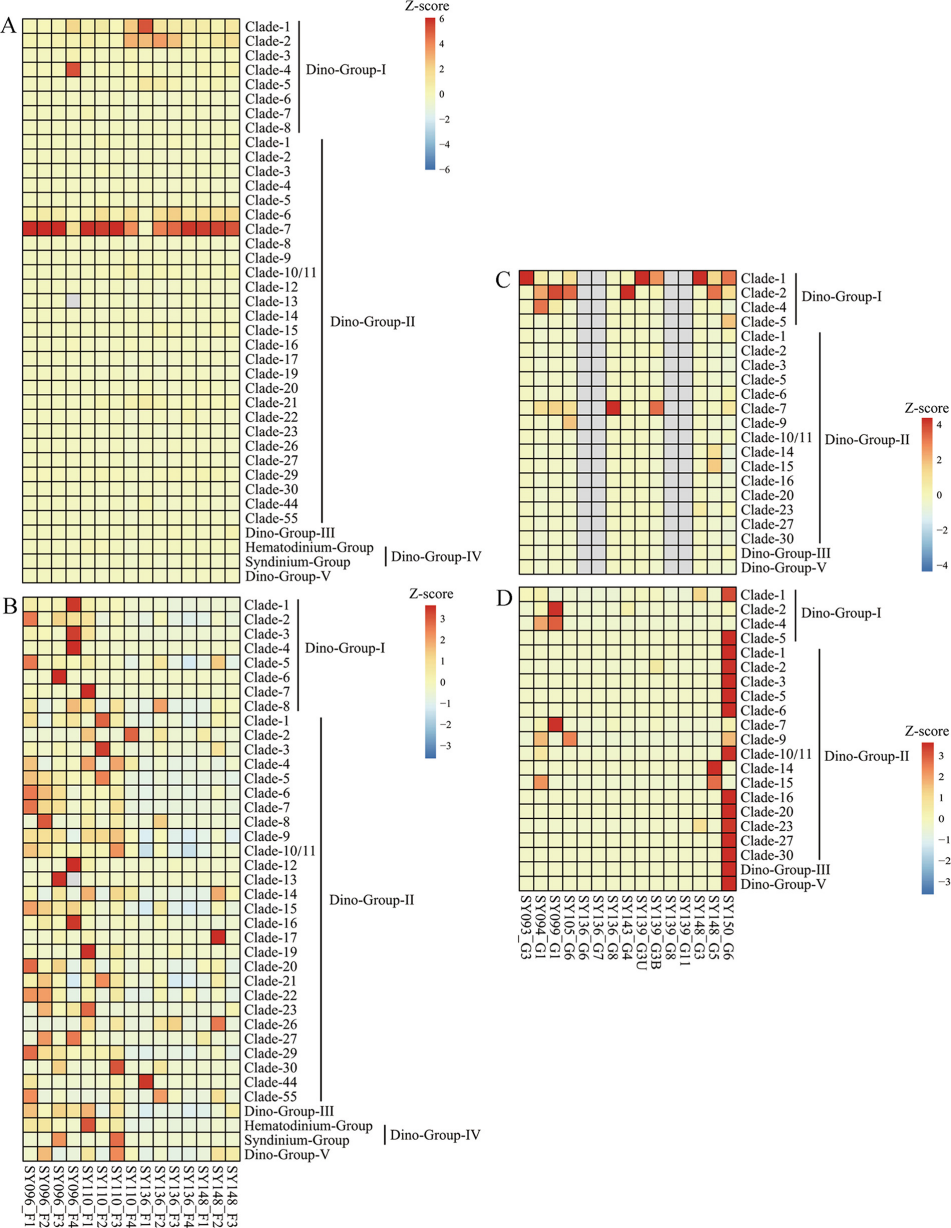
Z-score heat map boxes showing the relative abundances of different clades affiliated with the parasitic Syndiniales in fluids (A and B) and chimneys (C and D) in the southwest Indian Ocean. The data of panels A and C were normalized by column; the data of panels B and D were normalized by row.

Distinct distributions of different clades affiliated with parasitic Syndiniales in the fluids (Fig. 7A and C) and chimneys (Fig. 7B and D) were exhibited in the heat map. Dino-Group I and Dino-Group II were highly represented in the current study, with 8 and 27 clades, respectively, identified in fluids and 4 and 15 clades, respectively, identified in chimneys. In Dino-Group I, clades 1 and 2 accounted for higher proportions than other clades, while clade 7 was the predominant clade in Dino-Group II. Dino-Groups III, IV (mainly Hematodinium and Syndinium), and V accounted for lower proportions in all samples.

## DISCUSSION

The role of microeukaryotes as key components of the microbial loop has been investigated in hydrothermal vents, where numerous parasitic ecological types (31) and nutritional symbioses with chemosynthetic bacteria (32) have been revealed. However, microniche differentiations among different hydrothermal vents and between fluids and chimneys have been still largely unknown. In the current study, biogeographical distributions of diversified parasitic, heterotrophic, and symbiotic phylotypes were demonstrated, and a significant population divergence of parasitic Syndiniales was found between fluids and chimneys.

### Abundance and structure of microeukaryotes.

Hydrothermal vents harbor a flourishing microbiome (33, 34), especially the chemosynthetic microbial assemblages (35). We found that microeukaryote abundance ranged from ~10^4^ to 10^6^ copies/L in fluids and from ~10^4^ to 10^7^ copies/g in chimneys, and there was no significant difference among the three vents. This possibly suggested that geographical variations have little influence on the abundance of microprokaryotes. Previous studies also demonstrated that no obvious geographical difference was found for prokaryotic abundance in fluids (10^3^ to 10^5^ cells mL^−1^) or sediments (10^7^ to 10^8^ cells g^−1^) in the shallow-water hydrothermal systems (36) and deep-sea vents (37). In addition, the microeukaryotic abundance revealed in our study was much higher than that in the deep-sea pelagic waters (38), which might due to the increased food availability for microeukaryotes in hydrothermal fluids and chimneys (16, 39). Since this is the first report about microeukaryote abundance in the deep-sea vents, further proof will be needed to confirm their high abundance. It should be noted that although our primer set was designed specifically for protists (5 or more mismatches to prokaryotes [40]), it also could amplify metazoan sequences, as determined using the PR^2^ web primer tool (https://app.pr2-primers.org/pr2-primers/) (41). Therefore, the high number of gene copies in chimneys might be due to the unspecific amplification of metazoan groups.

Based on amplicon sequencing, alveolates, stramenopiles, and Rhizaria were found as predominant groups in our study, consisting with previous reports in the Guaymas Basin (20) and Mid-Atlantic Ridge (16) hydrothermal vents. The dominant vent-associated microeukaryote was Alveolata, specifically the group known as the Syndiniales, referred to as the marine alveolate (MALV) group. The Syndiniales are widely distributed in different samples in Longqi, perhaps due to their ability to colonize a wide range of ecological niches (42). The Radiolaria were another major group revealed in our study, and this group has been reported as one of the major players that export organic carbon to the deep sea (43, 44) and could host many living microorganisms, including dinoflagellates (45), to form a parasitic relationship. The high proportion and wide distribution of Syndiniales and Radiolaria occurring in our samples indicated the frequency of a parasitic metabolic status of microeukaryotes in the microbial ecosystems of hydrothermal vents. In addition, Apusomonadidae were widely distributed in the fluids of Kairei and Edmond, and this group has been found in seawater surrounding sulfidic chimneys and within vent bivalves (46) and in hydrothermal sediments with high metal concentrations in the Mid-Atlantic Ridge as well (47).

Ciliophora were retrieved from environmental sequencing as the main taxa for most chimney samples in our study, and this group has long been known to be the major grazers of bacteria and pico- or nano-sized eukaryotes in sediments (48) and bacterial mats near hydrothermal vents (16). Similar to previous studies on the hydrothermal vents in Guaymas Basin (49), Choanoflagellatea were also detected from chimney samples, and their high reproduction rates might allow these small flagellates to colonize different extreme habitats.

### Biogeography and environmental effects.

Clear biogeographical effects on the community composition of scaly-foot snails in the Kairei and Longqi vent fields have been revealed (50). In this study, microeukaryotic communities in the three different hydrothermal fields were distinct from each other, and the community diversities between Longqi and Kairei were significantly different in the fluids. This is very likely caused by the geochemistry of the fluids in each hydrothermal vent (51). A very high H_2_ concentration, a relatively high Si concentration, and a remarkably low CH_4_/H_2_ ratio might support a microbial ecosystem in Kairei that is distinct from those in other two vents (52). In addition, the high CH_4_ concentration found in both Longqi and Edmond might selectively support similar community structures.

In addition, the *in situ* hydrology of fluids also affects the distribution of microeukaryotes. Nitrate and silicate concentrations in the fluids exhibited a significant correlation with the fluid-associated microeukaryotic communities in our study. It was reasonable to assume that the fluid-associated microbes were exposed directly to the bulk seawater and subsequently affected by the water chemistry. The silicate concentration was much higher in Kairei, and this parameter has long been considered an important component of an early Earth prebiotic hydrothermal system and closely related to microbes in hydrothermal environments (53).

As for the chimneys, no obvious difference among the three vents was observed, suggesting that the microbial composition could not be explained by geographical proximity. Geographical distribution has been clearly observed for prokaryotes in different vents in the SWIR, which has been explained by different environmental temperatures (12). Discrepancies between the studies of Hu et al. (35) and Edwards et al. (54) could be due to the fact that microeukaryotes, an intermediate component in the microbial food web, might not be directly influenced by the water chemistry or minerals, unlike for prokaryotes. However, we did find distinct differences between chimneys coated with either Fe oxides or Fe-Si oxyhydroxides. The chimneys coated with Fe-Si oxyhydroxides tended to have a higher proportion of heterotrophic Fungi and Hilomonadea, while Hilomonadea were almost invisible in chimneys coated with Fe oxides. It was already known that minerals of chimneys serve as critical energy sources to support the metabolic growth of various chemolithoautotrophs (54, 55) and that chimney wall heterogeneity, such as variations in pore spaces, cracks, and fissures, would support different microbial populations (56). In turn, microbial biomineralization contributed to the formation of Fe and Si minerals in hydrothermal vents (53). However, the link between Fe-Si oxyhydroxides and the compositions/activities of microbes is currently unknown, and no direct proof regarding the effect of the minerals of chimneys on microeukaryotes is available. At this stage, we can only assume that chimneys coated with Fe oxides and Fe-Si oxyhydroxides support different prokaryotic groups, which in turn affects the composition of microeukaryotic communities.

### Potential trophic status in hydrothermal vents.

Microeukaryotes with a potentially different metabolic status were revealed in our study. Heterotrophic Ciliophora dominating in chimneys have been reported since they were important bacterivorous grazers in the hydrothermal fields (57). Oligohymenophorea and Spirotrichea classes accounted for the higher proportions in chimneys, and species affiliated with these groups may be especially suited to thrive within the venting environments (35). Plagiopylea and Euplotia specifically occurring in hydrothermal vents (58) were found in our study as well. They include species capable of thriving in low oxygen to suboxic environments with modified mitochondria (hydrogenosomes) and were known to form close associations with methanogens or bacteria (58). In addition, heterotrophic stramenopiles predominant in fluids showed a close affiliation with phylotypes from anoxic basins (20) and bathypelagic sediments (5) according to our phylogenetic analysis. They had higher relative sequence abundances in the Mariana Arc vent ecosystem (59), hydrothermally influenced water masses within the Okinawa Trough (60), and had been recognized as important bacterivores with a global distribution (61) and frequently observed in a deep-sea survey (62). The microeukaryotic phylotypes from the deep-sea hydrothermal vents revealed in this study were associated with those from various extreme environments, such as low-oxygen environments, anoxic basins, hydrothermal vents, and cold seeps. This might be because vents are reducing environments where oxygen has lower solubility at higher temperatures and thus share similar characteristics with those of low-oxygen environments and are subsequently suitable for the survival of certain microeukaryotic groups (47).

Despite the diversity and ubiquity of Syndiniales in the world’s oceans (2, 63), their ecological roles *in situ* have received little attention. In the present study, the presence of potential photoautotrophic groups (e.g., Chlorophyta and Streptophyta) in the fluids might have resulted from fast-sinking particles. Fast sinking has been considered the main reason for the presence of photoautotrophic cells in the dark ocean (64). The presence of well-preserved phytoplankton cells in the deep sea has been reported based on direct microscopy (65). Photoautotrophic groups could account for ca. 0.9% to 4.3% of the total RNA reads in the deep-sea waters (≥1,000 m) in the South China Sea (66). On the other hand, the mixotrophic life style (i.e., facultative heterotrophs) of these groups (66, 67) might be a reason as well. In addition, the autotrophic cyst-forming dinoflagellates Scrippsiella and Gymnodinium were found in our study. The dinoflagellate cysts in anoxic Delaware Inland Bay surface sediments have been identified at the RNA level (68). Cysts resistant to unfavorable conditions (69) or resting cysts as a part of life history (70) could survive in the dark deep sea for a long time.

Parasitism is an efficient strategy that facilitates the constant access of microeukaryotes to higher concentrations of organic materials (1). Parasitic protists with their hosts were densely populated in the hydrothermal vents in comparison to those in the cold deep-sea waters (31). The parasitic Syndiniales that thrive in venting systems revealed in our study might be attributed to the advantage of increased host availability in such extreme habitats. This group along with other microeukaryote lineages known to include parasitic species (e.g., ciliates, Amoebozoa, or Cercozoa), supports previous observations that parasitism is widespread and likely contributes to carbon turnover in deep-sea food webs (31). In addition, Syndiniales could prevent or delay energy transfer to higher trophic levels and to a certain extent cause carbon and nutrients to be diverted to the microbial loop through their influence on host traits and abundance (71, 72). Therefore, food web models should incorporate parasitic interactions for a better understanding of the role of parasitism in the carbon and nutrient cycling of the microbial ecosystems.

Syndiniales displayed a high level of diversity that might contribute to their successful colonization over a wide range of ecological niches (42). It was not surprising to find that clades 1 and 2 of Dino-Group I, which is affiliated with the parasitic Syndiniales, had a higher relative abundance in this study. Group I is specialized in the anoxic environments, particularly sediments (42), and these two clades had the capability to infect radiolarian Spumellaria (73). Dino-Group II, which is synonymous with Amoebophyra, was dominated by clade 7, which was more prevalent in aphotic layers and infects a broad range of marine dinoflagellates (42). Parasites could affect a number of trophic links and render the natural food web organization and the genetic composition of natural communities more complex (74). Therefore, the ecological significance of parasitism in the deep-sea hydrothermal vents deserves further investigation. If additional samples were available, specific marker genes, including oligonucleotide probes for groups of Syndiniales (75), could be applied in future studies to gain solid evidence of active parasitism in hydrothermal vents.

### Conclusions.

Our data further proposed that hydrothermal zones act as oases for parasitic microeukaryotes (31). Biogeographic distribution and related controlling factors of microeukaryotes among the three different hydrothermal fields were revealed, and the potentially diversified trophic status might help them to fulfill diverse roles in marine microbial ecosystems, especially in the complex deep-sea chemoautotrophic habitats. The distinct distribution of microeukaryotes, especially clades affiliated with parasitic Syndiniales, among different hydrothermal fluids and chimneys deserves further exploration to gain a deeper understanding of the trophic relationships and potential ecological function of microeukaryotes in the deep-sea extreme ecosystems.

## MATERIALS AND METHODS

### Sample collection.

Fluid and chimney samples were collected from three hydrothermal vents in the southwest Indian Ocean during cruise TS10 on R/V *Tan Suo Yi Hao* (Table 1; see Fig. S1 in the supplemental material), including eight fluid and four chimney samples from Longqi (37.78°S, 49.65°E), four fluid and four chimney samples from Kairei (25.32°S, 70.04°E), and three fluid and seven chimney samples from Edmond (23.88°S, 69.67°E).

The fluid samples were collected by a rosette sampler coupled with a conductivity-temperature-depth (CTD) instrument and a MARP (miniature autonomous plume recorder). Samples were first identified by turbidity anomalies using a MARP with an optical backscatter sensor for turbidity measurement. Hydrothermal fluid samples were confirmed by measurement of high concentrations of CH_4_, a commonly used tracer for hydrothermal fluids (76). The concentrations of inorganic nutrients (i.e., nitrate, silicate, nitrite, and phosphate) of the fluids were analyzed with an autoanalyzer (QuAAtro; BLTEC Co., Ltd.). For molecular analysis, fluids were sequentially filtered through 3- to 0.22-μm-pore-size polycarbonate filters (47 mm; EMD Millipore, Billerica, MA, USA). All of the filters were immediately flash frozen and stored at −80°C until further analysis.

As for chimney samples, they were collected by the mobile industrial robots (MiR) arm of the manned submersible *Shen Hai Yong Shi* and placed in hermetic containers at −80°C until further analysis. *In situ* hydrographical parameters (i.e., depth and location) were recorded during sampling by the submersible. The chimneys continuously interact with seawater, resulting in the oxidative weathering and alteration of various metal oxides such as Fe oxides. The characteristics of chimney samples were recorded at the scene of grasping.

### Nucleic acid extraction, PCR amplification, and sequencing.

The total DNA for the fluid samples (~5 L) was extracted from the 0.22-μm-pore-size polycarbonate filters with a PureLink genomic DNA kit (Invitrogen, Carlsbad, CA), in accordance with the manufacturer’s instructions. The total DNA for the chimney samples (~0.5 g) was extracted using a PowerSoil DNA isolation kit (MO BIO Laboratories, Inc., Carlsbad, CA, USA), according to the manufacturer’s protocol. The DNA was quantified with a Qubit 2.0 fluorometer (Life Technologies, USA), and the quality was checked via gel electrophoresis.

DNA was amplified using the FastStart high-fidelity PCR system (Roche) with the following universal primers: TAReuk454FWD1 [5′-CCAGCA(G/C)C(C/T)GCGGTAATTCC-3′] and REV3 [5′-ACTTTCGTTCTTGAT(C/T)(A/G)A-3′] (77), to target the V4 domain of the 18S rRNA gene. The PCR was performed with an initial denaturation step of 95°C for 3 min, followed by 32 cycles of 95°C for 30 s, 55°C for 30 s, and 72°C for 1 min, after which there was a final extension step of 72°C for 5 min. A negative control of double-distilled water was also performed during amplification in order to avoid reagent contamination. The paired-end sequencing of the amplicons was performed with an Illumina HiSeq PE250 sequencer (Novogene Co., Ltd.; https://en.novogene.com).

### Quantitative PCR.

The abundance of the 18S rRNA gene in fluid and chimney samples was quantified using the StepOnePlus quantitative PCR (qPCR) system (Applied Biosystems, Inc., Carlsbad, CA, USA). Each qPCR mixture comprised 10 μL 2× SYBR Premix Ex Taq II (TaKaRa Bio, Inc., Shiga, Japan), 0.3 μM euk345f/Euk499r primers (75), 2 μL DNA as the template, 0.4 μL ROX reference dye, and water to a total of 20 μL. The qPCRs and calibrations were performed as described by Zhu et al. (40). Triplicate qPCRs were performed for each sample with efficiencies of 94.51%, and the gene copy number was normalized to the quantity of the gene. As a positive control, a linear plasmid, which was constructed using the amplified PCR products and a TOPO-TA vector cloning kit (Invitrogen), was used.

### Bioinformatics analysis.

After sequencing, primary processing of raw fastq files and demultiplexing of paired-end sequences were performed using QIIME 2 (78). Trimming, primer sequence removal, sequence denoising, paired-end merging, filtering of chimeric sequences, singleton removal, and sequence dereplication were completed with DADA2 (79). The representative sequences were picked and then compared with the PR^2^ database version 19 (80). Taxonomy assignment of amplicon sequence variants (ASVs) that were not affiliated with microeukaryotes (including bacteria and archaea, as well as metazoan and plastidial sequences) were further removed (78). A filtered ASV table for each sample was generated with QIIME 2.

### Network analysis.

To explore the cooccurrence patterns between taxa, network analysis was conducted by calculating the correlations. A similarity matrix was first generated by inputting a typical ASV matrix file, and then the correlation matrix, *R* value, and *P* value were calculated using the corr.test function in the “psych” v2.1.9 package (81) of R v3.5.3. The threshold value was calculated to reflect the interaction between species, and the value within the range of occor.p > 0.01|abs (occor.r) < 0.6 was converted to 0 to obtain a comma-separated values (CSV) file, which was input into Gephi v0.9.2 to construct the networks with a Spearman’s *ρ* of >0.6 and a false discovery rate-adjusted *P* of <0.05, and was visualized using R packages (vegan and igraph) and the software Gephi 0.9.2 (82).

### Phylogenetic analysis.

The phylogenetic affiliations of the most abundant ASVs (top 50 in sequence abundance) for the respective fluid and chimney samples were determined by construction of maximum-likelihood (ML) trees with a bootstrap value of 1,000 replicates using MEGA6 (83) with the general time reversible model and the TN93+G and K2+G methods for 1,000 bootstrap iterations for the fluid and chimney samples, respectively.

### Statistical analysis.

The box plot of diversity indexes, the heat map, and the bubble plots of relative abundances of taxa were generated using the “ggplot2” v3.3.5 packages (84) in R version 3.5.3. Venn diagrams were generated to show shared ASVs among sampling sites using R package “limma” v3.50.3 (85). The nonlinear multidimensional scaling (nMDS), based on the Bray-Curtis similarity index, was calculated with PRIMER 5 (Plymouth Marine Laboratory, West Hoe, Plymouth, UK) (86) to show the distribution pattern of microeukaryotic communities. An analysis of similarities (ANOSIM), based on ASV sequence abundance and the diversity index (i.e., Shannon and Simpson indexes), was conducted with Paleontological Statistics (PAST) version 3 (87) to test whether there was a significant difference in the microbial communities among the various sampling sites. A redundancy analysis (RDA) was performed with Canoco V5.0 to identify a possible differentiation of the communities under the constraint of environmental factors and to assess correlations between environmental variables and community structures. The phylogenetic data were Hellinger transformed, environmental variables were logarithm transformed, and the effects of collinearity (variance inflation factor > 10) were removed.

### Data availability.

All of the 18S rRNA gene sequences obtained from this study have been deposited in the National Center for Biotechnology Information (NCBI) Sequence Read Archive (SRA) under accession number PRJNA754718. The ancillary data and sample table with the corresponding codes used for the sequence libraries and paper were deposited in figshare (https://figshare.com/) under doi 10.6084/m9.figshare.19665993. All scripts were deposited in figshare under doi 10.6084/m9.figshare.19666128.
