# Supplementary material for: Biogeography and Population Divergence of Microeukaryotes Associated with Fluids and Chimneys in the Hydrothermal Vents of the Southwest Indian Ocean
Source: Microbiol Spectr. 2022 Sep 19;10(5):e02632-21. doi: 10.1128/spectrum.02632-21 (PMC9603758; doi:10.1128/spectrum.02632-21)
Supplement: Supplemental file 1 — Table S1; Fig. S1 to S3. Download spectrum.02632-21-s0001.pdf, PDF file, 0.3 MB [file spectrum.02632-21-s0001.pdf]

Biogeography and population divergence of micro-eukaryotes associated with fluids and chimneys in the hydrothermal vents of the Southwest Indian Ocean

Yue Zhang[1#], Ning Huang[1#], Hongmei Jing[1,2,3*]

[1]*CAS Key Laboratory for Experimental Study under Deep-sea Extreme Conditions, Institute of Deep-sea Science and Engineering, Chinese Academy of Sciences, Sanya 572000, China;*

[2]*HKUST-CAS Sanya Joint Laboratory of Marine Science Research, Chinese Academy of Sciences, Sanya, China;*

[3]*Southern Marine Science and Engineering Guangdong Laboratory (Zhuhai), China*

Running Title: Micro-eukaryotes in Hydrothermal vents

Keywords: Micro-eukaryotes, Hydrothermal vents, High-throughput sequencing, Parasitic, Distribution

# Contributed equally.

[*]For correspondence**:** E-mail: hmjing@idsse.ac.cn

Supplementary materials

Table S1 The coordinate, sequencing information and diversity index of the fluids and chimney samples collected from the hydrothermal vents in the Southwest Indian Ocean.

Samples	Lon (°E)	Lat (°S)	Original Reads	Quality Reads	ASVs	Shannon	Simpson
SY096_F1	49.6483	37.7822	75,769	71,482	569	6.17	0.96
SY096_F2	49.6483	37.7822	13,918	12,551	257	6.00	0.96
SY096_F3	49.6483	37.7822	67,585	61,598	695	6.26	0.95
SY096_F4	49.6483	37.7822	68,393	64,429	508	7.07	0.98
SY110_F1	48.6493	37.7840	75,063	68,372	550	6.24	0.94
SY110_F2	48.6493	37.7840	50,022	45,338	260	5.09	0.88
SY110_F3	48.6493	37.7840	67,812	61,813	593	5.66	0.92
SY110_F4	48.6493	37.7840	70,177	63,899	400	4.32	0.86
SY136_F1	70.0398	25.3200	24,187	12,672	75	2.98	0.74
SY136_F2	70.0398	25.3200	75,213	67,024	466	4.35	0.81
SY136_F3	70.0398	25.3200	78,031	69,940	362	2.96	0.68
SY136_F4	70.0398	25.3200	76,322	69,287	237	2.63	0.60
SY148_F1	23.8780	69.5968	49,252	44,088	316	4.18	0.89
SY148_F2	23.8780	69.5968	69,168	64,004	346	5.16	0.91
SY148_F3	23.8780	69.5968	52,716	48,404	400	4.74	0.84
SY093_G3	49.6483	37.7822	68,845	52,273	57	4.24	0.89
SY094_G1	49.6495	37.7841	65,373	37,218	81	3.29	0.77
SY099_G1	49.6503	37.7841	63,015	44,734	13	3.17	0.86
SY105_G6	49.6498	37.7837	63,155	45,685	78	4.48	0.89
SY136_G6	70.0397	25.3200	16,957	12,733	30	3.56	0.83
SY136_G7	70.0397	25.3205	60,234	44,447	50	1.71	0.38
SY136_G8	70.0400	25.3207	6,754	4817	26	3.04	0.81
SY143_G4	70.0397	25.3204	30,840	18,552	83	4.14	0.88
SY139_G3U	23.8780	69.5971	65,334	47,897	67	4.51	0.90
SY139_G3B	23.8780	69.5971	64,086	43,917	128	3.78	0.74
SY139_G8	23.8780	69.5969	63,273	47,833	29	3.78	0.87
SY139_G11	23.8780	69.5969	47,372	31,467	27	1.32	0.34
SY148_G3	23.8780	69.5968	67,710	51,227	53	4.52	0.93
SY148_G5	23.8780	69.5968	65,981	47,622	203	5.56	0.94
SY150_G6	23.8781	69.5968	65,814	50,545	264	6.45	0.96

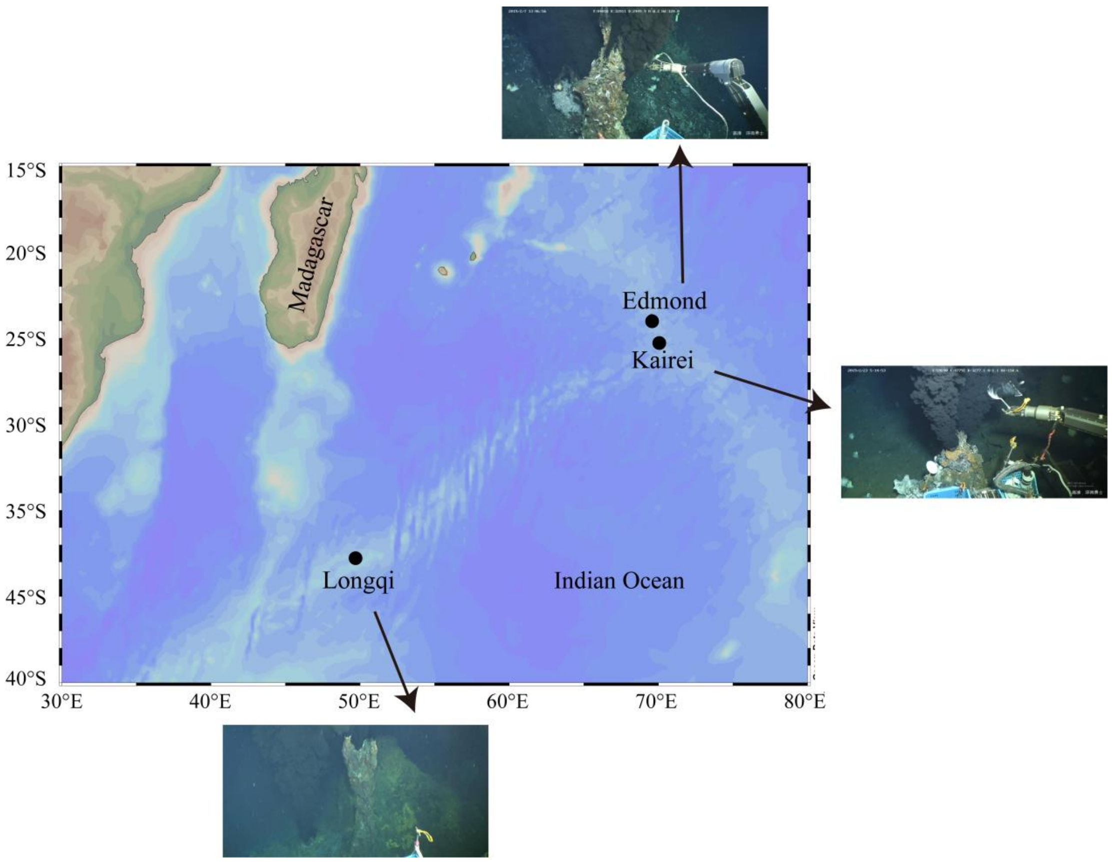

Figure S1 Map of the hydrothermal vents in the Southwest Indian Ocean.

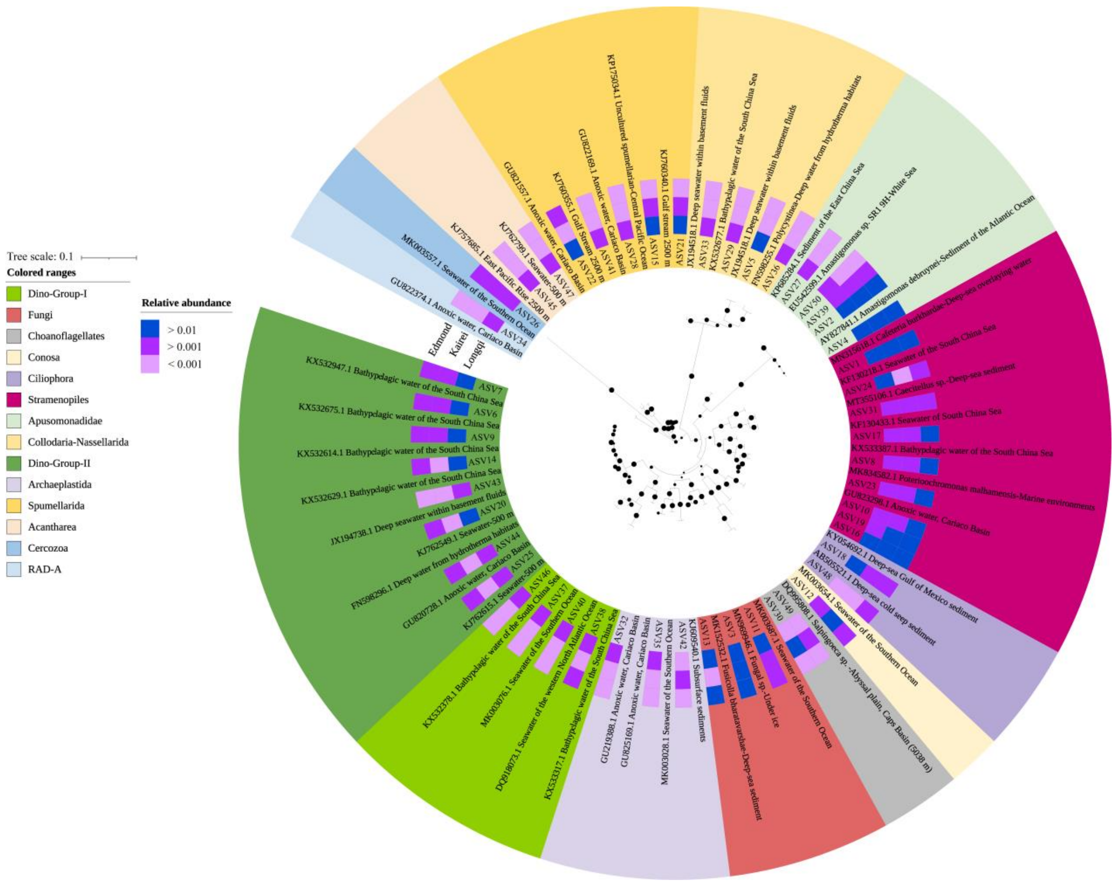

Figure S2 Maximum-likelihood phylogenetic tree constructed with top 50 ASVs for fluids in different hydrothermal vents in the Southwest Indian Ocean. A bootstrap value was calculated 1,000 times. Color scale represents the distribution of ASVs, which were calculated based on the percentage of sequences for each ASV out of the total sequences obtained.

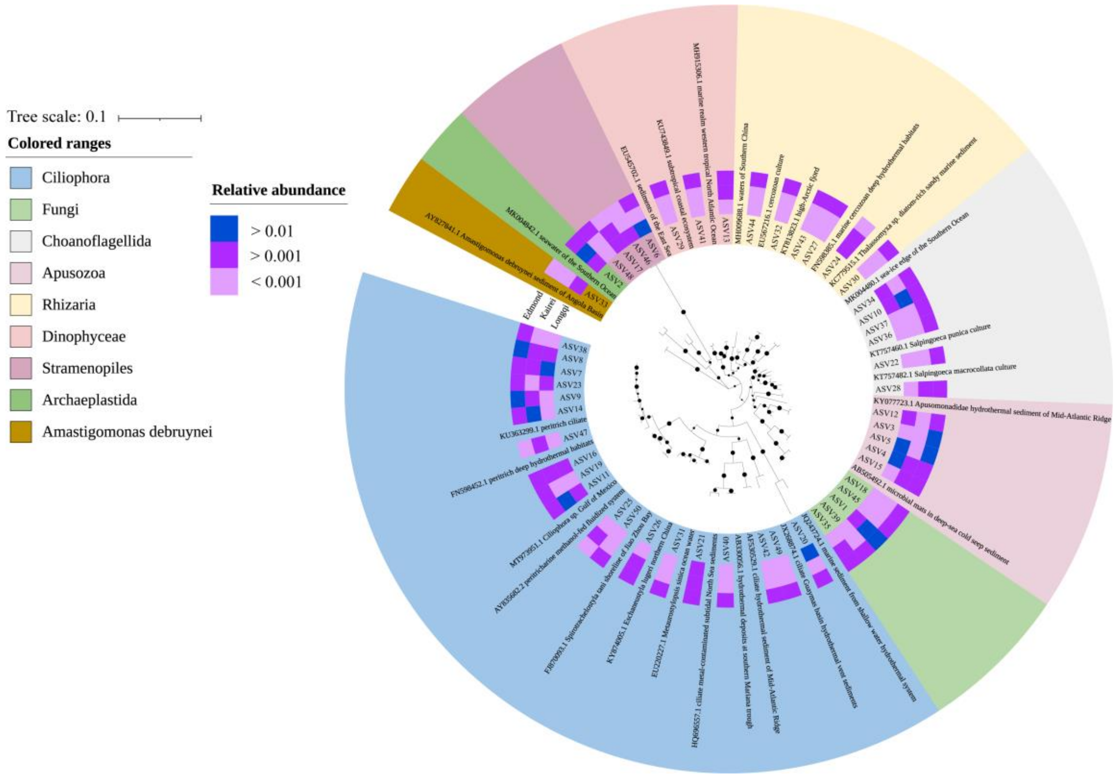

Figure S3 Maximum-likelihood phylogenetic tree constructed with top 50 ASVs for chimneys in different hydrothermal vents in the Southwest Indian Ocean. A bootstrap value was calculated 1,000 times. Color scale represents the distribution of ASVs, which were calculated based on the percentage of sequences for each ASV out of the total sequences obtained.
